# Characterisation of ground motion recording stations in the Groningen gas field

**DOI:** 10.1007/s10950-017-9725-6

**Published:** 2018-01-03

**Authors:** Rik Noorlandt, Pauline P. Kruiver, Marco P. E. de Kleine, Marios Karaoulis, Ger de Lange, Antonio Di Matteo, Julius von Ketelhodt, Elmer Ruigrok, Benjamin Edwards, Adrian Rodriguez-Marek, Julian J. Bommer, Jan van Elk, Dirk Doornhof

**Affiliations:** 1 Deltares, P.O. Box 85467, 3508 AL Utrecht, The Netherlands; 2 Shell Global Solutions International B.V, Kessler Park 1, 2288 GS Rijswijk, The Netherlands; 3 Geotomographie GmbH Germany, now at School of Geosciences, University of the Witwatersrand Johannesburg, WITS, 2050 South Africa; 4 Royal Netherlands Meteorological Institute (KNMI), Utrechtseweg 297, 3731 GA De Bilt, The Netherlands; 5 Department of Earth, Ocean and Ecological Sciences, University of Liverpool, Liverpool, L69 3GP UK; 6 Charles E Via, Jr., Department of Civil and Environmental Engineering, Virginia Tech, Blacksburg, VA 24061 USA; 7 Civil & Environmental Engineering, Imperial College London, London, SW7 2AZ UK; 8 Nederlandse Aardolie Maatschappij B.V, Schepersmaat 2, 9405 TA Assen, The Netherlands

**Keywords:** Shear-wave velocity, Field measurements, MASW, Cross-hole tomography, Seismic cone penetration test, Shallow geology, Lateral heterogeneity

## Abstract

The seismic hazard and risk analysis for the onshore Groningen gas field requires information about local soil properties, in particular shear-wave velocity (*V*_S_). A fieldwork campaign was conducted at 18 surface accelerograph stations of the monitoring network. The subsurface in the region consists of unconsolidated sediments and is heterogeneous in composition and properties. A range of different methods was applied to acquire in situ *V*_S_ values to a target depth of at least 30 m. The techniques include seismic cone penetration tests (SCPT) with varying source offsets, multichannel analysis of surface waves (MASW) on Rayleigh waves with different processing approaches, microtremor array, cross-hole tomography and suspension P-S logging. The offset SCPT, cross-hole tomography and common midpoint cross-correlation (CMPcc) processing of MASW data all revealed lateral variations on length scales of several to tens of metres in this geological setting. SCPTs resulted in very detailed *V*_S_ profiles with depth, but represent point measurements in a heterogeneous environment. The MASW results represent *V*_S_ information on a larger spatial scale and smooth some of the heterogeneity encountered at the sites. The combination of MASW and SCPT proved to be a powerful and cost-effective approach in determining representative *V*_S_ profiles at the accelerograph station sites. The measured *V*_S_ profiles correspond well with the modelled profiles and they significantly enhance the ground motion model derivation. The similarity between the theoretical transfer function from the *V*_S_ profile and the observed amplification from vertical array stations is also excellent.

## Introduction

Induced earthquakes due to gas production in the Groningen field in the northern Netherlands has prompted the development of seismic hazard and loss estimation models in order to allow risk-informed decision-making with regard to mitigation options. A key element of the seismic hazard and risk models for the Groningen field is a ground motion prediction model to estimate surface motions due to each possible earthquake scenario. The ground motion model for the Groningen field is comprised of predictive equations for spectral accelerations and peak ground velocity at a reference rock horizon (located at about 800 m depth) and non-linear frequency-dependent amplification functions reflecting the dynamic response of the overlying soil layers (Bommer et al., 2017).

**Fig. 1:**
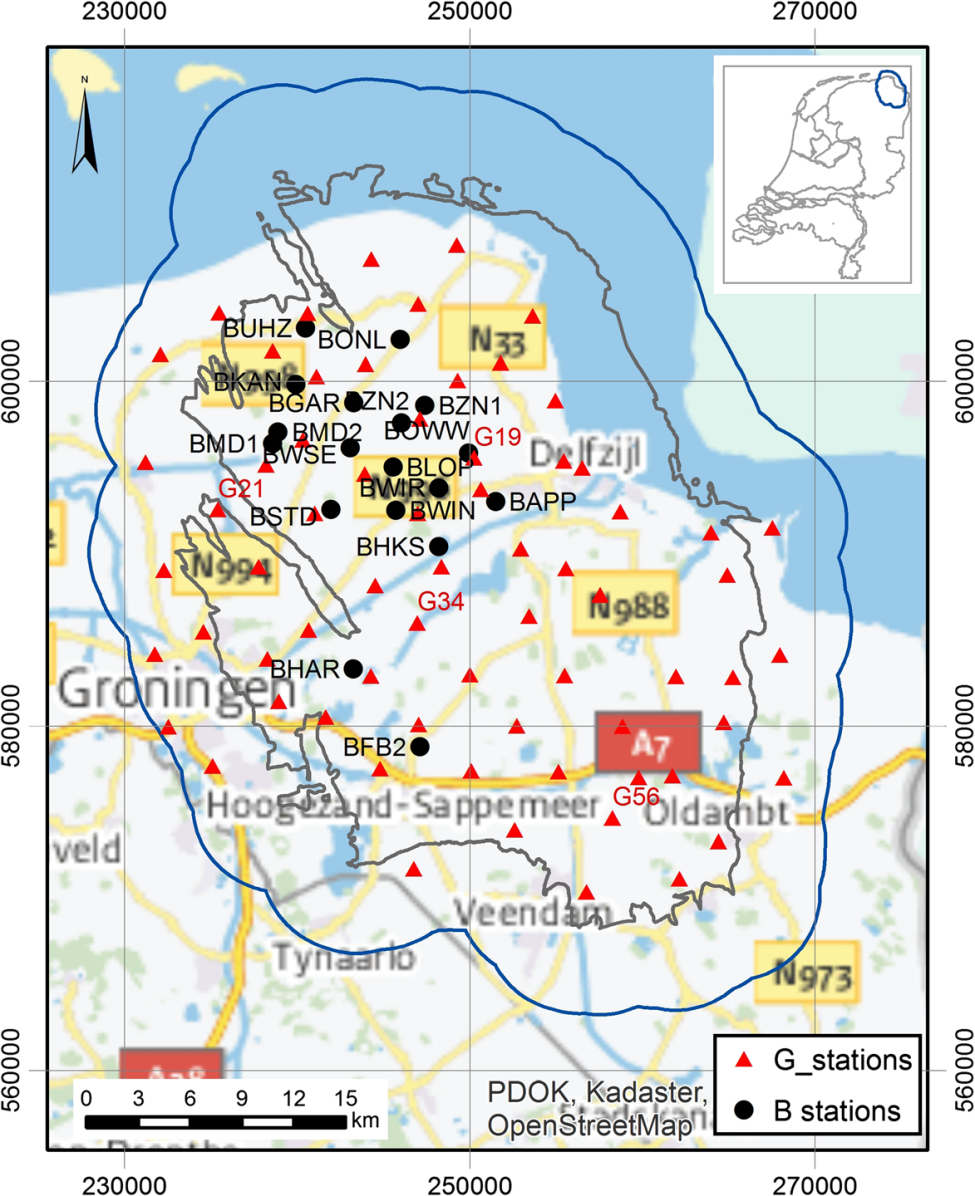
Location of recording stations in the Groningen field in the north of the Netherlands: 18 surface stations and 68 vertical array stations. Labels are shown for all 18 surface stations (coded B) characterised in this study and for the vertical array stations (coded G) from the examples in this paper (Figs. 16 and 18). Grid coordinates refer to the Dutch Ordnance System. The inset shows the location of the gas field in the northern part of the Netherlands

The ground motion model derivation has benefited from a database of recordings of ground motions obtained from accelerograph and borehole geophone networks installed in the Groningen field. The location of the stations is shown in Fig. 1. The first stage of the model building process is to deconvolve the recorded surface motions to the reference rock horizon. The uncertainty in this process is greatly reduced by the accurate characterisation of dynamic properties of the soil column, particularly in the uppermost tens of metres that exert the strongest influence on the site response. Although an excellent velocity model of the Groningen field has been constructed using measurements at depths from below about 50 m, the near-surface portion of the profiles are inferred from lithological profiles with shear-wave velocities (*V*_S_) assigned based on available seismic CPT measurements (Kruiver et al., 2017a). To refine the profiles at the locations of the ground motion recording stations, in situ *V*_S_ measurements were made using a variety of borehole and non-invasive techniques. Challenges encountered in this work include the fact that in several cases it was not possible to perform the measurements in very close proximity to the location of the recording stations. The paper describes how these tests were conducted and the procedures followed to reconcile the different measurements to construct the final profile for each station.

The measured near-surface profiles also served to demonstrate that the geologically-derived *V*_S_ profiles provide a very good approximation to the field conditions. Empirical transfer functions at the recording stations obtained from inversions of the surface recordings (Edwards et al., 2013) agree remarkably well with those calculated using the measured *V*_S_ profiles (Bommer et al., 2017). These comparisons not only confirm the reliability of the inferred velocity profiles for the whole field but also vindicate the assumption of 1D vertical wave propagation implicit in the site response analyses.

No direct *V*_S_ measurements have been made at the borehole stations, but interval velocities have been calculated from recordings at these locations and these also show excellent agreement with the inferred profiles for the same locations. The theoretical transfer functions for these profiles, calculated at the surface and 200 m depth, are similar to the surface-to-borehole spectral ratios of earthquake records.

## Methods and setup

### Overview

The shallow subsurface in the Groningen region is of heterogeneous composition as a result of the relatively recent formation. Although site amplification as a result of induced earthquake is present in Groningen to larger depths, the maximum depth of investigation was limited to 30 m. This depth of investigation is not related to *V*_S30_ (time-averaged *V*_S_ over the top 30 m), but forms a good balance between fieldwork effort and added value of detailed *V*_S_ profiles to this depth. The geological setting is described in detail in Kruiver et al. (2017b) and references therein and summarised in this section. The sedimentary infill is influenced by two recent ice ages and by sea level fluctuations. The Elsterian glaciation produced deep subglacial features known as ‘tunnel valleys’, which were filled with sands and clays of the Peelo Formation. These tunnel valleys were buried by younger sediments. The second glaciation (Drenthe Substage of the Saalian glacial) produced the till sheet that is present in part of the region. The ridge-and-valley topography is still present in the relatively flat landscape. The region was not covered by ice sheets during the last ice-age (Weichselian). During that period, a widespread superficial blanket of eolian sand (the so-called cover sand) that formed in many places marks the top of the Pleistocene deposits. The northern part of the Netherlands borders the North Sea. During interglacial periods with relatively high sea levels, a large part of Groningen formed the coastal plain of this sea. The most recent Holocene deposits typically consist of stacked vertical sequences of tidal clays and sands that are often thinly bedded and are intermittent with peat layers. The Holocene sediment thickness varies from ~ 20 m in the northern part to being absent in the southern part of the region. Due to the presence of various intersection channel systems, namely the Pleistocene tunnel valleys and Holocene tidal channels, the subsurface is very heterogeneous. From the very large number of borings and from the geology, we can infer that infilled channels are present. It is, however, impossible to know the exact location of all individual channels.

In order to characterise the subsoil below the recording stations and considering the level of heterogeneity to be expected, the *V*_S_ measurements were to be located as close to the stations as possible. The stations are generally located in barns of farms in the rural areas and in public buildings (e.g. town halls) and houses in villages. Therefore, it was not always possible to locate all measurements in close vicinity of the stations. The distance between the station and the test site varied between 40 and 600 m, with an average distance of 150 m.

Four different *V*_S_ techniques were applied at the station locations. This section provides a short description of acquisition and processing for each of the methods. The survey setup is summarised in Table 1. Although the methods are routinely used in site characterisations (e.g. Garofalo et al. 2016a, b), we have implemented several adjustments to either improve the acquisition or the processing and interpretation of results. Suspension P-S logging (Ohya et al., 1984; Ogura et al., 1989) was applied unsuccessfully during this survey campaign, probably due to the combination of the borehole construction, grouting and the local geological setting. Therefore, these data were not further processed and interpreted.

**Table 1:** Summary of survey setup for the four different shear-wave methods

	SCPT	MASW	Microtremor array	Cross-hole tomography	Suspension P-S logging
Source	Wooden beam and sledgehammer at 1.1, 5, 10, 15 and 20 m from SCPT truck	Accelerated weight drop ‘Impacter’	Ambient noise	Borehole source of type BIS-SH	Hammer source in tool
Receivers	3-component accelerometer in SCPT cone	96 to 120 planted vertical 4.5 Hz geophones	Planted geophones of MASW array	3-component geophone string of 7 units with spacing of 1.0 m	Two 3-component hydrophone receivers in tool separated by acoustic damping tubes
		12 planted vertical 1 Hz geophones			
Remark	Vertical sample interval max 1.0 m and coinciding with stratigraphical transitions	T-shaped array with geophone spacing of 2.0 or 3.0 m (4.5 Hz) and 4.0 m (1 Hz).	Recording of 70-80 × 32 s (120 × 16 s at one site)	L-shaped array of 3 boreholes with the source the corner of the L and the receivers in borehole at 10 (short leg of L) or 25 m (log leg of L). Boreholes are lined with blind liners	In one of the boreholes for cross-hole tomography
Depth of investigation	20–30 m	15–50 m	10–50 m	30 m	30 m
Lateral averaging	~ 2 m	Up to ~ 200 m		~ 1 m	~ 1 m
Vertical resolution	High, except shallow part	Medium, decreasing with depth	Medium, decreasing with depth	High	High

### SCPT

**Fig. 2:**
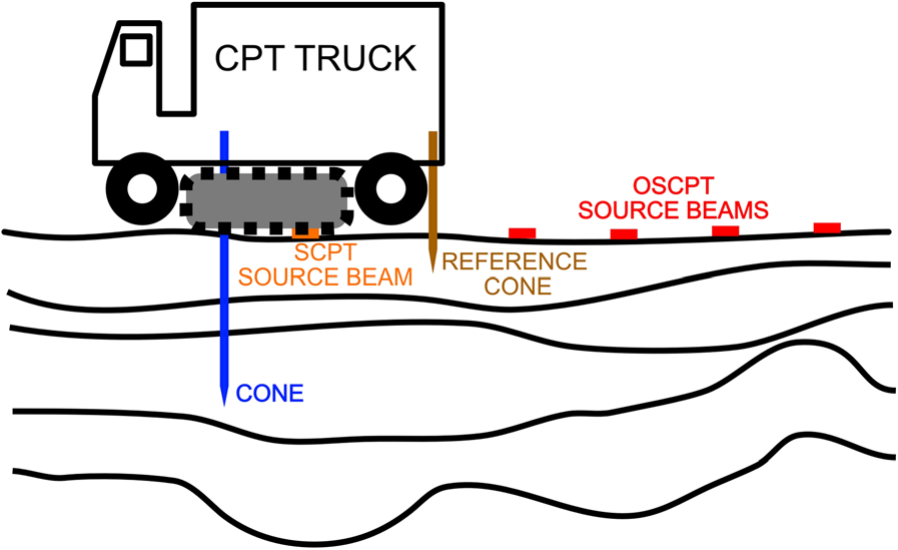
Schematic illustration of offset SCPT setup (not to scale)

Seismic cone penetration tests consist of a normal CPT with a geophone or accelerometer contained in the cone. The cone is penetrated into the soils and stopped at defined depth intervals for a *V*_S_ measurement. Shear waves were generated at the surface by striking a 10-kg sledgehammer on opposite sides of 2.5 m hardwood beams. Typically, the cone penetration is stopped every 1.0 m and the source is located ~ 1 m from the entry point at the surface (Butcher et al., 2005). In the Groningen case, alternations between peat, clay and sand occur at irregular intervals that are often smaller than 50 cm. In order to correctly sample the *V*_S_ of for each individual stratigraphy, the depth intervals at which the cone was stopped were determined by a normal CPT that preceded the SCPT. For example, when an 80-cm thick peat layer was present between 1.7 and 2.5 m on top on of sand, a *V*_S_ measurement was performed at 1.7 and 2.5 m instead of at 2.0 and 3.0 m. In this way, the peat *V*_S_ was not contaminated by the much stiffer sand below it. Sources at offsets of 5, 10, 15 and 20 m were added to the standard 1.1 m source to gain insight in short-spaced lateral variations in *V*_S_ (Fig. 2). This is referred to as offset SCPT (OSCPT). Coupling of the wooden beams to the ground was increased by sand bags. At each source location, three shots were performed and stacked at each depth, both from the left and right sides of the beam. The maximum target depth of SCPTs was 30 m, but in some cases the measurement was terminated earlier (e.g. at ~ 20 m) when the cone could not be penetrated further upon reaching the maximum capacity of the truck.

**Fig. 3:**
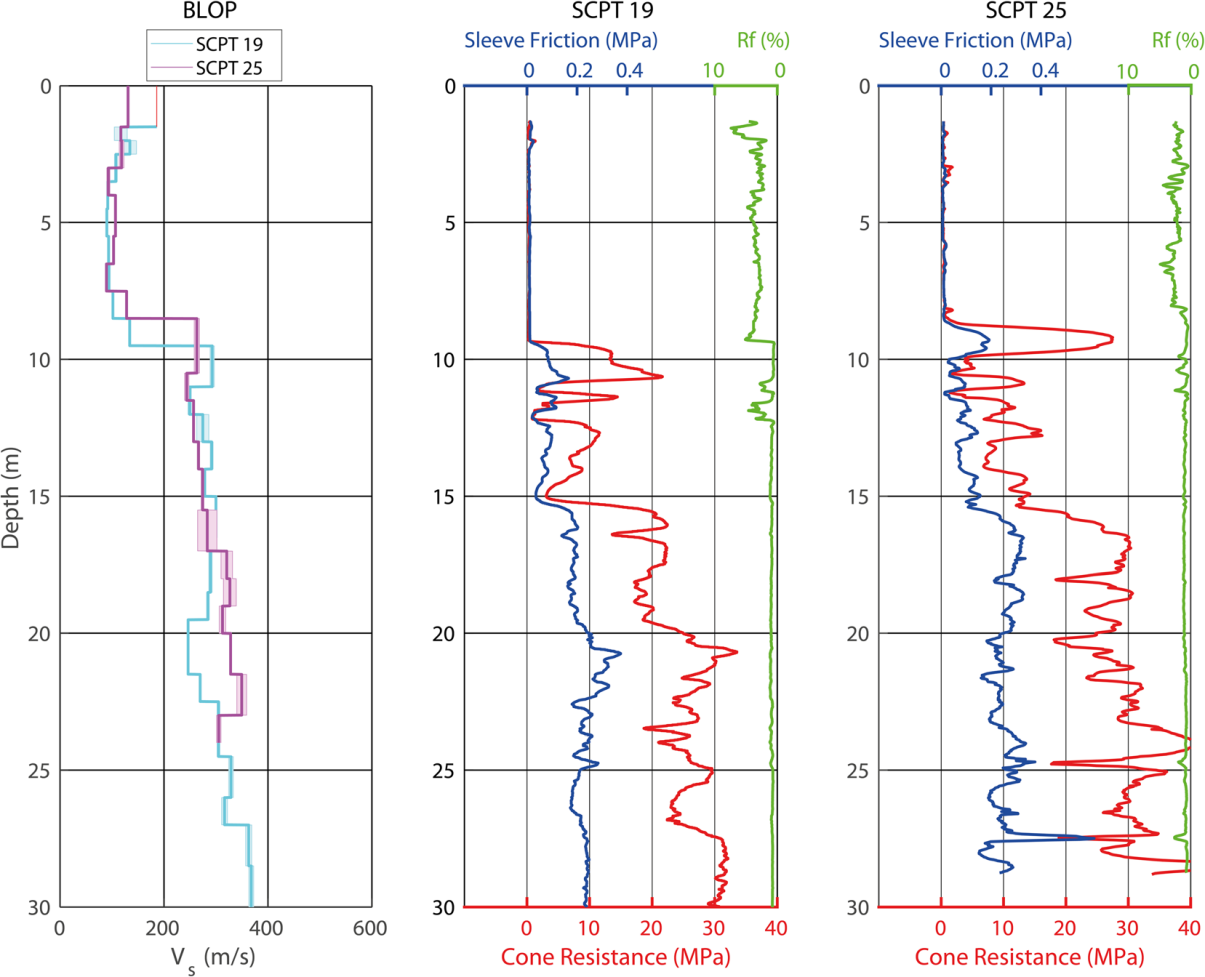
CPT and SCPT data for station BLOP. Left: *V*_S_ profiles of SCPT 19 (blue) and SCPT 25 (purple). The shaded band indicates the results from the left and the right blow. Middle and right: CPT soundings of SCPT 19 and SCPT 25, with cone resistance in red, sleeve friction in dark blue and friction ratio Rf in green

**Fig. 4:**
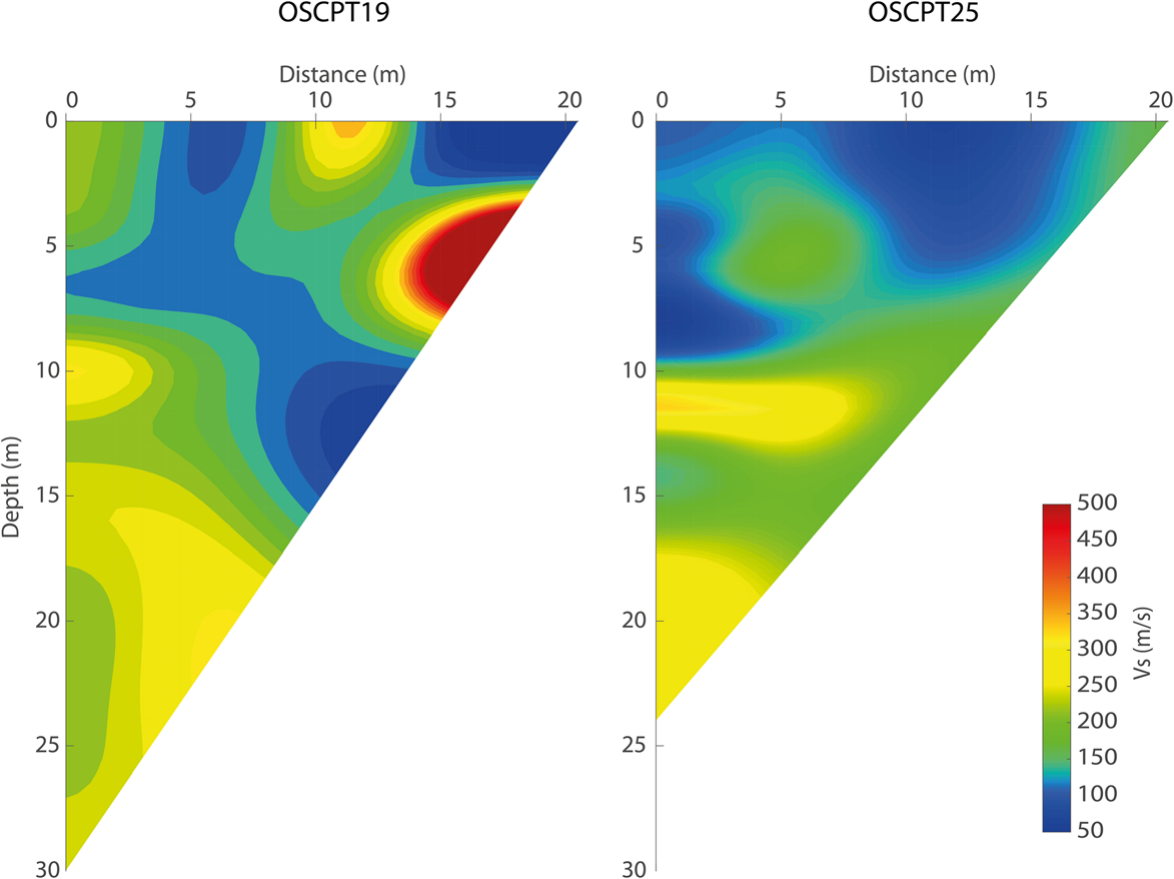
OSCPT result for SCPT19 (left) and SCPT25 (right) for station BLOP

SCPT data were processed using the BCE SC3-RAV 2015 seismic data analysis software (Version 15.0.1-June 2015). This software allows semi-automatic interval time picking using cross-correlation of the wave trains of subsequent test depths. The algorithm uses a simple ray tracing principle based on a horizontal stratigraphy model to determine the travel path length to calculate the interval shear-wave velocities. The left and right shots from the shear-wave source were processed separately. The traces for the OSCPT had to be hand-picked due to the lower signal-to-noise ratio for the larger offsets. The model subsurface from the OSCPT data was discretised using a grid of nodes, with a node distance of 0.5 m. For each of the nodes, the optimum *V*_S_ was determined by minimising the misfit defined as the root-mean-square (RMS) between the modelled travel times and the measured travel times. The ‘fast marching method’ (Sethian, 1999) was used to calculate the modelled travel times of seismic waves from source to receivers. For the optimisation, the Fresnel ray-path approach (Watanabe et al., 1999) was used. An example of CPT soundings and SCPT *V*_S_ profiles for station BLOP is given in Fig. 3. The distance between the (S)CPTs is ~ 80 m. The CPT soundings show transitions at 8–9 m (Naaldwijk clay to Boxtel sand and Drente-Gieten clayey sand), 11.5–12 m (Drente-Gieten clayey sand to Peelo fine sand) and 15.5–16.5 m (Peelo fine sand to Peelo medium sand). The transitions in two nearby (S)CPTs do not occur at the exactly same depth, illustrating the heterogeneity of the geology. The transition between Holocene and Pleistocene Formations at 8–9 m depth can be clearly observed as a jump in the SCPT *V*_S_ profiles. The effect of the transition between different lithoclasses within the Peelo Formation at 15–16 m depth is rather subtle. The OCPTs images (Fig. 4) are much smoother than an individual SCPT *V*_S_ profile. Figure 4 consistently shows the Holocene/Pleistocene *V*_S_ transition and the Boxtel/Drente-Gieten Formations on top of the Peelo Formation. The Boxtel Formation can be cemented very locally, giving rise to relatively high *V*_S_ values. The OSCPT images show that even on very short (~ metre) spatial scales the values of *V*_S_ and the transition depths vary.

### MASW and microtremor array method

**Fig. 5:**
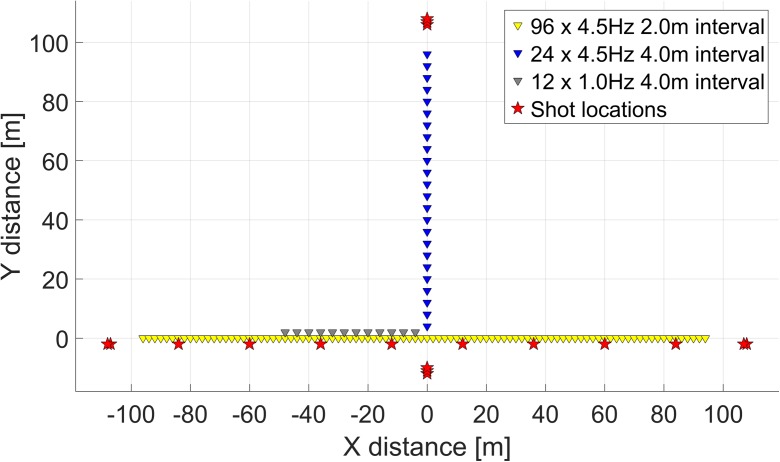
Survey setup for MASW and microtremor array with planted geophones. Triangles represent geophone locations, and stars represent shot locations

The MASW method uses the dispersive behaviour of surface waves, i.e. the fact that the different frequencies of the wave signal travel with different speeds, to derive *V*_S_ profiles with depth (Park et al. 1999). The dispersion of a wave can be determined using multiple receivers that record the passage of a surface wave. The surface wave itself can be actively generated for the analysis, for example with a hammer blow or weight drop, or can be of ambient origin, like traffic or ocean noise. If active source and ambient noise recordings contain different frequency ranges, they might be combined to increase the depth range and resolution. However, while the prominent source direction is known in an active acquisition, in ambient noise acquisition it is unknown beforehand. Therefore, we acquired the seismic data using T-shaped arrays, with different sets of geophones, as visualised in Fig. 5 and summarised in Table 1. The main line consisted of 96 4.5-Hz geophones (72 in the first locations). To obtain lower frequency content 12 1-Hz geophones were placed parallel to the main line. Perpendicular to the main line 24 4.5-Hz geophones were placed for directionality analysis. For the active data acquisition an accelerated weight drop source (‘Impacter’) was used, at the shot locations indicated in Fig. 5.

Our analysis of MASW and microtremor array data is based on records of Rayleigh waves. Generally, the lowest frequency of both microtremor array and MASW data is 2 to 4 Hz and the maximum usable wavelength under the assumption of a homogeneous medium ranges between ~ 40 and 200 m. Given the heterogeneous and layered subsurface of the Groningen region, with low *V*_S_ layers and *V*_S_ values decreasing at certain depths, the theoretical wavelength at a certain frequency does not represent the true penetration depth. Although wavelengths of about 200 m were observed, the inversion of the data showed that typical penetration depth was in the order of 10 to 50 m.

**Fig. 6:**
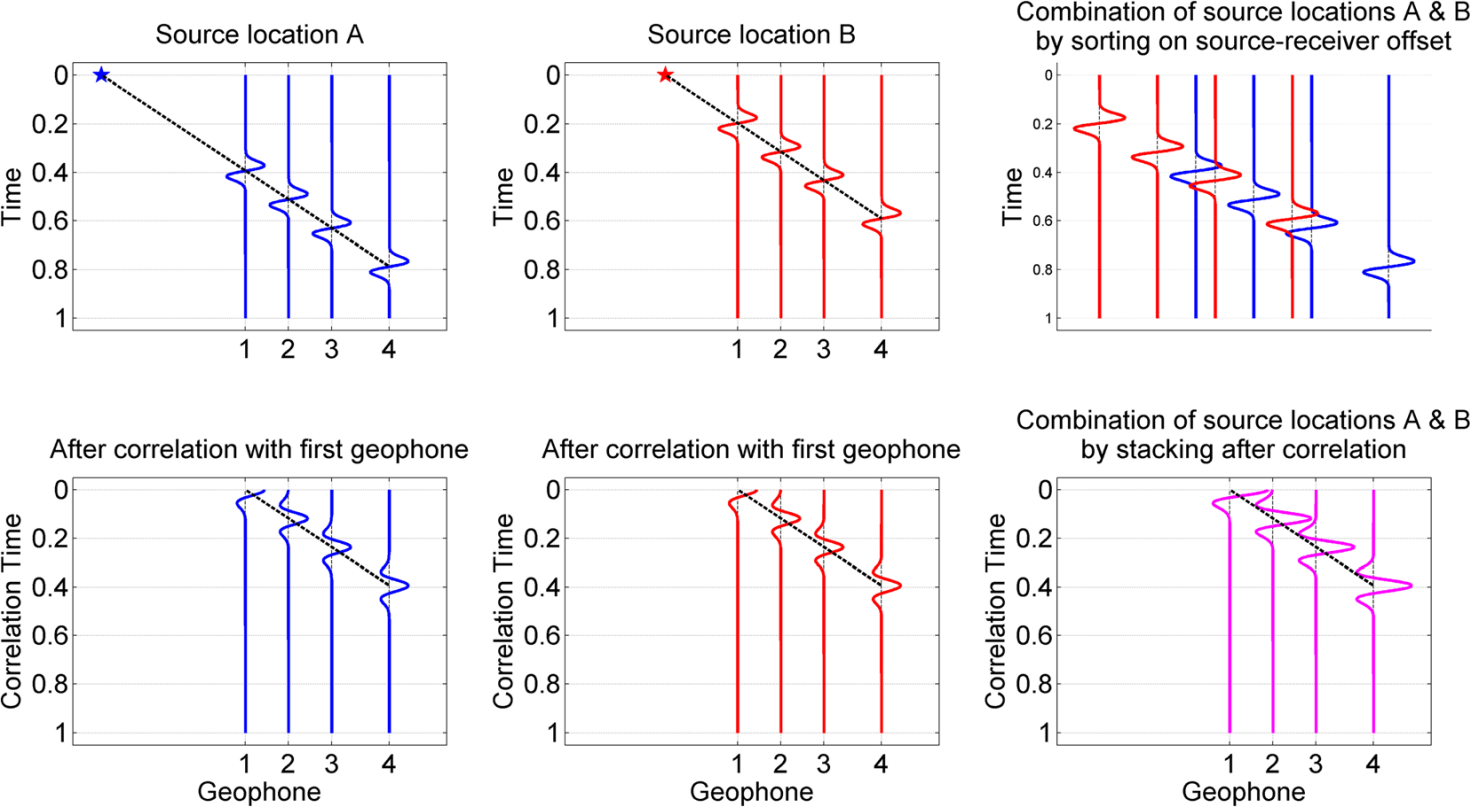
Sorting and pre-processing of MASW data to obtain offset gathers (top) and geophone correlation gathers (bottom) to prepare for CMPcc binning

The dispersion analysis of the MASW data was done in two ways, making use of the different source locations. The first method focuses on getting the highest resolution dispersion plot for the whole line, while the second method focuses on determining multiple (lower resolution) dispersion plots along the array to detect heterogeneity within the array. The basic idea behind the methods is sketched in Fig. 6. The static array was used in two ways to combine multiple shot locations. In the method sketched in the top row, the geophones are sorted on source-receiver offset to get a densely-sampled virtual record. This is referred to as an offset gather. For the main line the sampling was improved from 96 channels at 2 m interval to 192 channels at 1 m interval by shifting the source by 1 m. In this way, the spatial sampling interval is halved and the amount of data is doubled. This procedure was possible because the Impacter (accelerated weight drop) produced a repeatable signal.

**Fig. 7:**
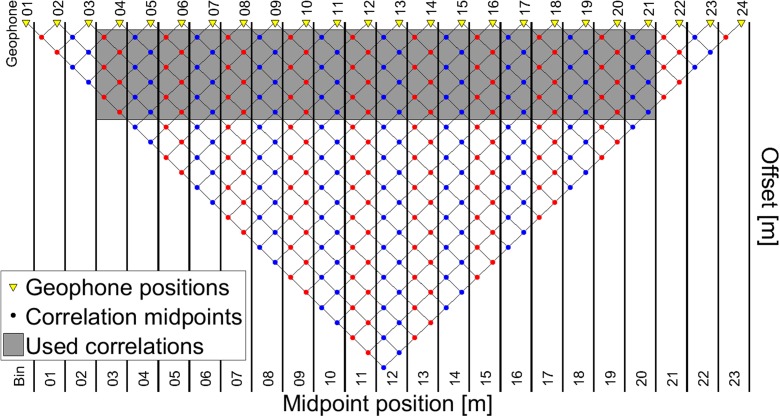
Schematic representation of CMPcc binning. Geophones are represented by yellow triangles, and the correlation midpoints are indicated by dots. The correlations within the grey zone are used for analysis. Only bins with a sufficient number of midpoints are used (bins 3 to 20 in this example) and fixed offset range (6 geophones in this example)

The second method focuses on determining heterogeneity and is sketched in the bottom row of Fig. 6. The influence of the source-receiver offset is removed by correlating the recordings from different receivers, for subsequent sources, and stacking these correlations over the sources. Virtual records along the array are generated using different correlation pairs. The dispersion behaviour of the virtual records is determined using the common midpoint cross-correlation approach (CMPcc) approach (Hayashi and Suzuki, 2004). Although 96 geophones can be combined to form a maximum of 4560 correlation pairs, approximately one third of the combinations were used. This is to ensure equal numbers of pairs in each CMPcc bin (Fig. 7). For visualisation purposes, the array has been reduced to 24 geophones in this figure, but the same principle applies to the 96 geophones of our array. Correlations are binned based on the midpoint of the geophone pairs being correlated. The different offsets between the correlation pairs within a bin are used to determine the dispersion for each bin. A minimum number of correlation pairs within a bin is required to estimate the dispersion with sufficient confidence. Therefore, the first and last bins cannot be used for dispersion plotting because they contain an insufficient number of pairs spanning too limited an offset. This applies to the first two and last three bins in Fig. 7. Correlations pairs with too large an offset were excluded as well, because the purpose of the CMPcc analysis was to investigate heterogeneity. This procedure ensures that each bin contains the same number of correlation pairs. Therefore, changes in dispersion can be attributed to differences of ground properties, rather than to resolution differences between the bins. Our CMPcc bins contained 24 geophone pairs per bin, spanning an offset between 2 and 48 m. Dispersion plots were generated from the CMPcc bins for waves that were travelling from ‘left-to-right’ as well as for ‘right-to-left’ travelling waves. The best quality dispersion plot was selected for analysis.

**Fig. 8:**
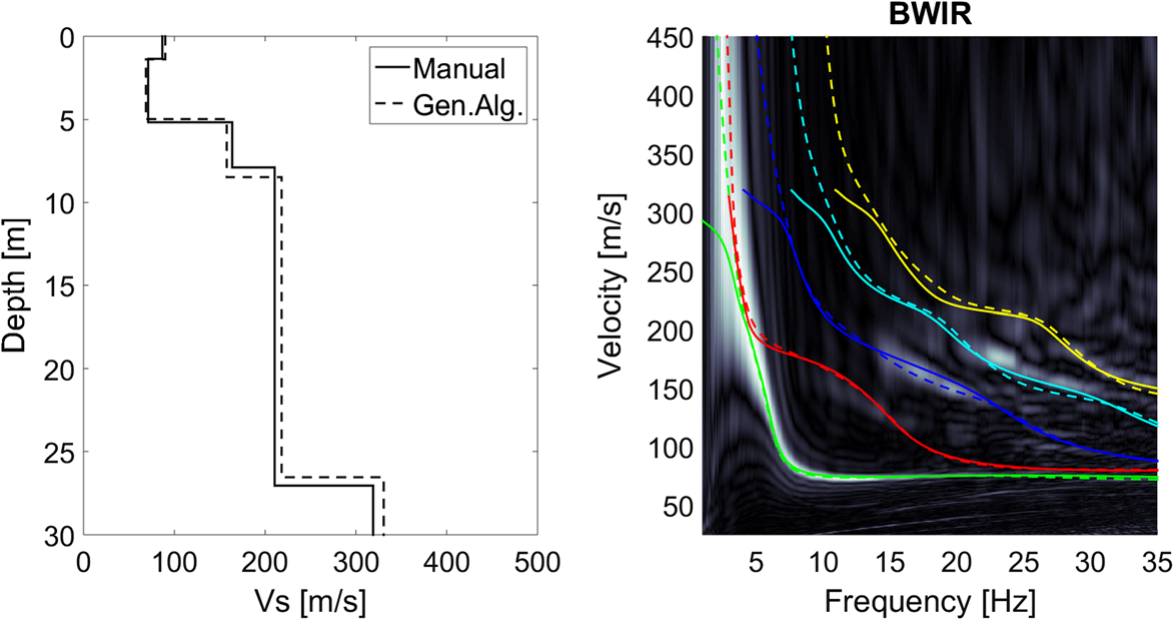
MASW result for station BWIR. Left: *V*_S_ profiles from the manual optimisation (solid line) and the genetic algorithm (dashed line). Right: dispersion plot showing the energy in the velocity- frequency domain in grey scale and the theoretical dispersion curves for the *V*_S_ profiles of the left panel for the manual optimisation (solid line) and the genetic algorithm (dashed line). The fundamental mode is shown in green; the higher modes in red, blue, cyan and yellow

The dispersion plots found by the classic MASW and the CMPcc approach were inverted to derive *V*_S_ models and investigate the *V*_S_ variation along the profile. The inversion algorithm searches the model space to find the *V*_S_ profile with the minimum misfit between the modelled dispersion curve and the measured energy on the dispersion plot. The most likely *V*_S_ model for each data set was determined with in-house software by manual optimisation and by applying an automated genetic algorithm. The full array was processed both manually and automatically. The CPMcc gathers were processed automatically. For the manual optimisation, the SCPT V_S_ model was used as a starting model. The emphasis of this exercise was to obtain the *V*_S_ model that fits all modes and the particular shapes of the modes, such as curvatures at certain frequencies. The genetic algorithm automatically generates numerous *V*_S_ model realisations, each associated with a modelled dispersion curve. The best *V*_S_ model from the manual optimisation was used to define the search space of the automatic algorithm. The goodness of fit was defined by the energy of the dispersion plot along the modelled dispersion curve. After a number of iterations, the genetic algorithm converges to a group of likely models. The best *V*_S_ model is chosen from this group based on the best goodness of fit. Figure 8 shows an example of a dispersion plot of the full line array of 96 geophones and resulting *V*_S_ profiles obtained with the two inversion approaches. Generally, the two approaches result in the same pattern of *V*_S_, but the transition depths and *V*_S_ values of the individual layers vary between the methods. The theoretical fundamental and the higher modes corresponding to the *V*_S_ profile for both methods are shown in the dispersion plot. Only the fundamental mode is used in the goodness of fit definition in the genetic algorithm. All modes are considered in the visual inspection of the fit between the model and the data in the manual approach. The maximum depth of reliable *V*_S_ information was determined by a sensitivity analysis of the deeper layers to changes in depth and *V*_S_ during the manual procedure. The maximum reliable depth for *V*_S_ varied between 10 m for a site with a very thick layer of low *V*_S_ to ~ 50 m for sites with stiffer soils.

**Fig. 9:**
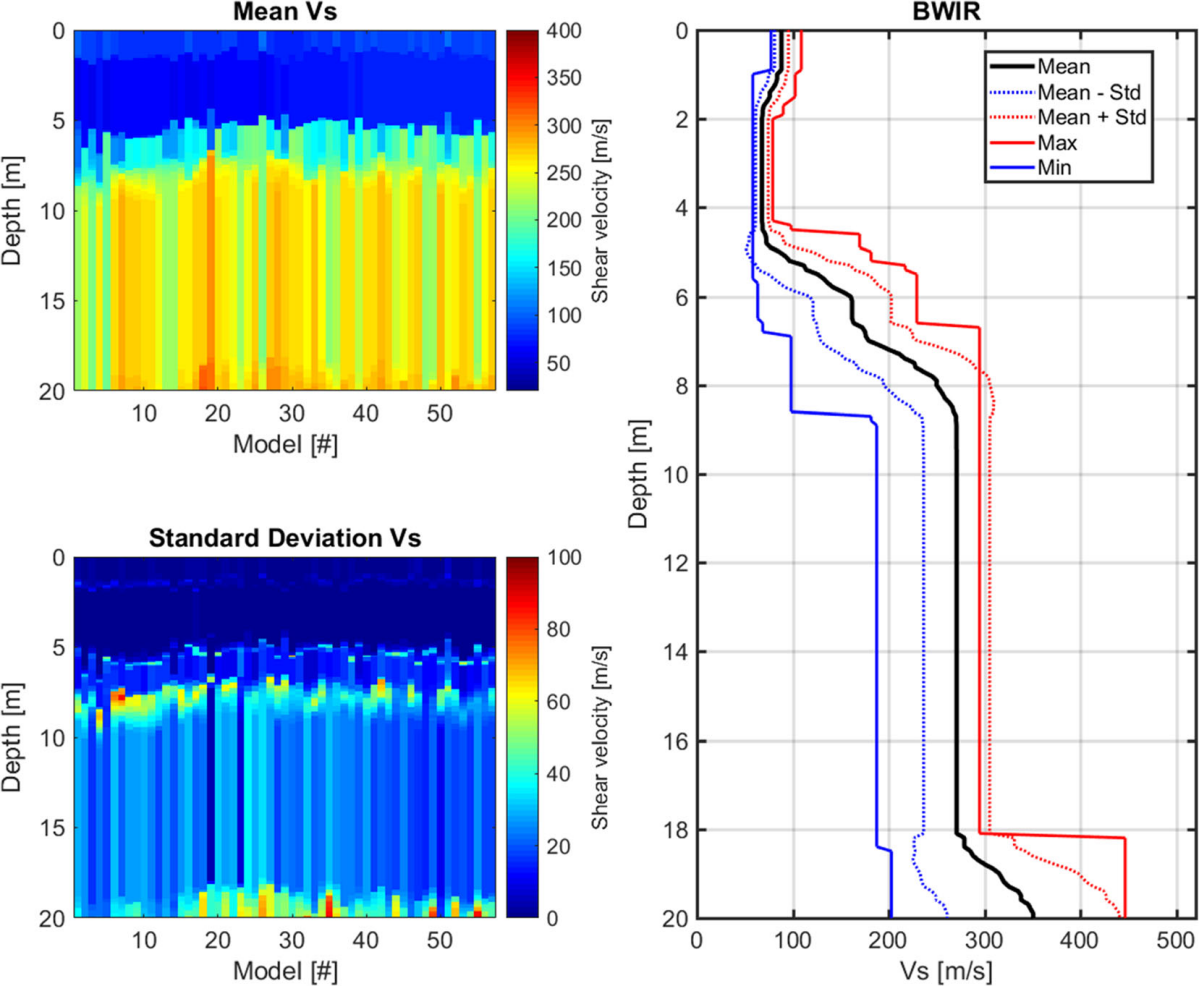
CMPcc result for station BWIR. Top left: mean of best *V*_S_ models for the 72 CMPcc gathers along the line. Bottom left: standard deviation of best *V*_S_ models for the 72 CMPcc gathers along the line. Right: best fit curve of the 72 models with the maximum and minimum value observed in the whole CMPcc and the standard deviation from all models

The CMPcc result for station BWIR is shown in Fig. 9. The top left panel shows the best *V*_S_ model for each of the 72 CMPcc gathers as determined using the genetic algorithm. The *V*_S_ profile resulting from each CMPcc gather is represented by a colour-coded column. The panel of 72 columns does not represent a 2D *V*_S_ profile, because of the large overlap in data between in the CMPcc models (Fig. 7). However, the plot is indicative of variation in thickness and *V*_S_ values of the individual layers along the full array of ~ 200 m length. The transition between Holocene and Pleistocene deposits at ~ 8–9 m (in SCPT) varies between ~ 4 and 9 m. The bottom left panel of Fig. 9 shows the standard deviation of the group of best models for each CMPcc gather. The standard deviation varies laterally as well and is generally higher for deeper layers. This means that *V*_S_ for deeper layers is less well constrained. The aggregated results of the CMPcc analysis are shown in the right panel of Fig. 9. The transitions between the model layers appear to be smeared relative to the standard MASW interpretation which shows sharp transitions (Fig. 8).

Passive or ambient-vibration-based surface wave methods record background vibrations emanating from ocean wave activity, atmospheric conditions, wind effects, traffic, industrial, construction activities, etc., which collectively are referred to as microseisms. Examples of application of microseism techniques can be found in Yong et al. (2013). Typically, microseisms with frequencies below 1 Hz have natural origins, whereas those above 1 Hz are largely due to human activities (Okada, 2003). As frequencies below 1 Hz are difficult to generate by active sources, microseisms can help increasing the bandwidth at the low end and therefore the microtremor array technique will usually extend the depth of investigation of MASW.

**Fig. 10:**
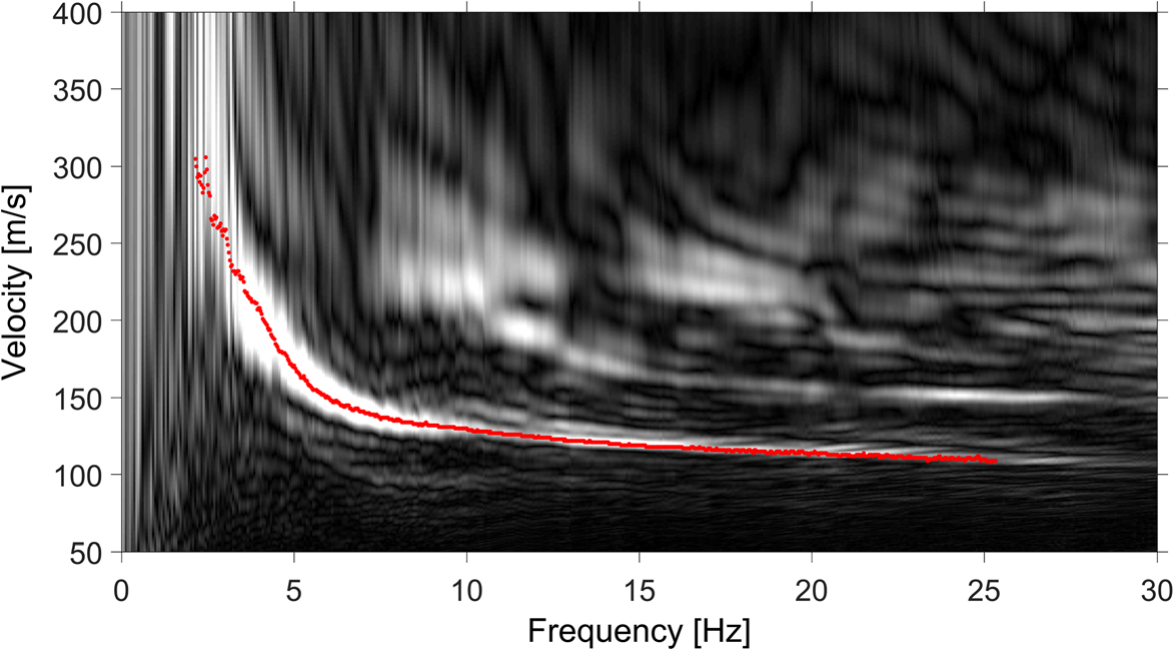
Dispersion curves determined with the ESAC method applied on the passive data at BGAR (red dots) plotted on top of the (black and white) dispersion plot determined for the active data using the MASW method

The microtremor array experiment was conducted to test whether the MASW data could be enhanced by including ambient noise data. The microtremor array data were acquired using the same planted geophone arrays as the MASW with a source (Table 1). However, this time ambient noise was recorded. At each site, between 70 and 80 recordings were made, each with duration of 32 s at a sample interval of 2 ms. The microtremor array data were processed using the extended spatial autocorrelation (ESAC) technique (e.g. Mulargia and Castellaro, 2013). ESAC is based on the spatial autocorrelation (SPAC) method of Aki (1957). A drawback of the ESAC method is that only a single dispersion curve is determined, most likely the fundamental mode, but the method is considered more suitable for microtremor array measurements than frequency-wavenumber spectrum (fk) methods (Ohori et al., 2002). Figure 10 shows the dispersion information obtained from the microtremor array and active source data acquired at station BGAR. The dispersion curve from the microtremor array measurements (red dots in Fig. 10) agrees well with the fundamental mode in the dispersion plot of the MASW data (greyscale dispersion plot of Fig. 10). However, the microtremor array data do not significantly extend the bandwidth of the dispersion data.

During the processing of the microtremor array dataset, it was not possible to extract surface wave dispersion data from the ambient noise acquired at sites BAPP and BHKS. The MASW results indicate the presence of a thin, low-velocity (*V*_S_ ~ 50 m/s) surface layer at these sites. Lateral variations in thickness and *V*_S_ of top layers have a relatively large influence on the dispersion in the EPAC procedure and can affect the whole *V*_S_ model. The dispersion energy in the dispersion plot therefore becomes less reliable or impossible to define on the dispersion curve. The MASW data for these two stations showed good quality dispersion plots. The maximum resolved depth of stations BAPP and BHKS was limited to ~ 10–20 m (instead of 30 m) due to the presence of the very low-velocity layer at the top.

**Fig. 11:**
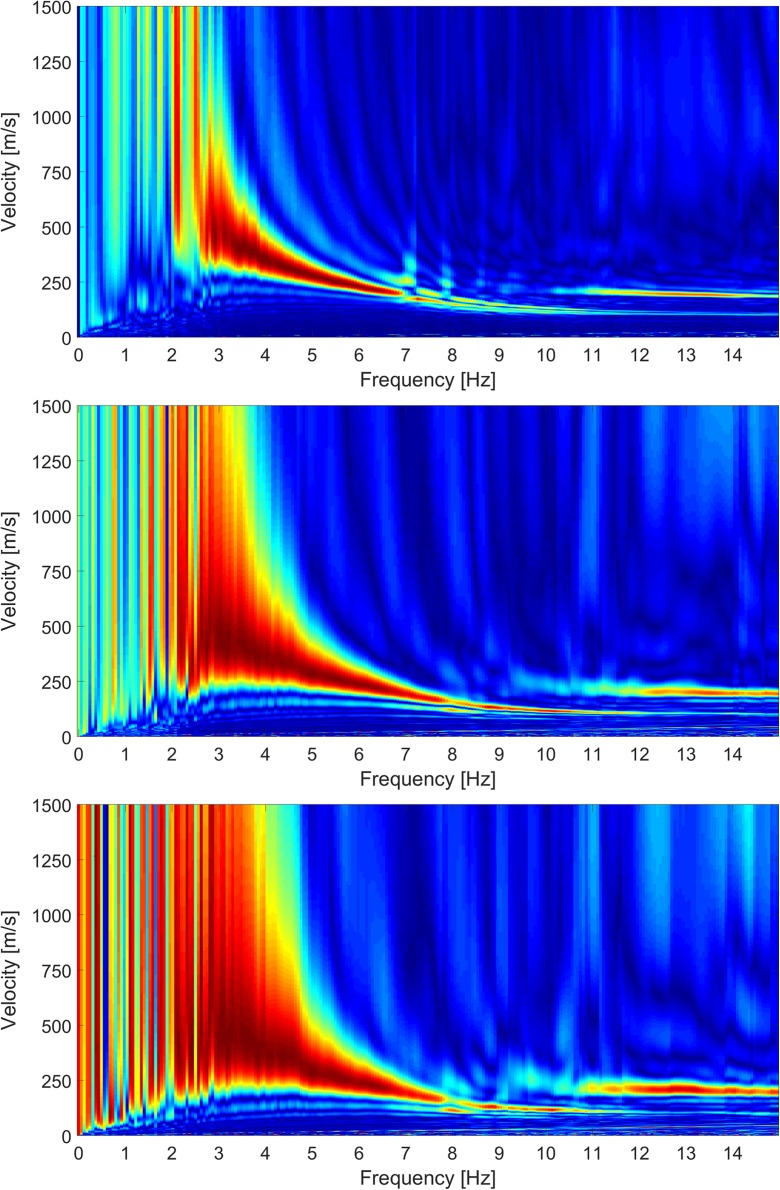
Comparison of dispersion plots for station BKAN. Top: 4.5 Hz array of all 96 geophones. Middle: 4.5 Hz array of 12 geophones at corresponding locations of 1 Hz array. Bottom: 1 Hz array of 12 geophones

Another means to extend the low-frequency range of the MASW is to use low-frequency geophones. We installed 1 Hz geophones which partially coincided with the 4.5 Hz array (Fig. 5). Because of the high costs of these geophones, only 12 were available, which creates a very short array. Nonetheless, the dispersion plots of the 1 Hz array and the 4.5 Hz array are compared in Fig. 11. The effect from the number of geophones in the array is clear from the top and middle panel, both for the 4.5 Hz geophones. Reduction of the number of geophones results in smeared dispersion energy. The 1 Hz array shows more low-frequency energy than the corresponding 4.5 Hz array (bottom versus middle panel). In the current setup, however, the quality is insufficient to be able to extend the dispersion curve to lower frequencies. The quality could be improved by using a low-frequency source such as a low-frequency shear wave vibrator and by deploying more 1 Hz geophones at larger distances between the geophones. Ambient noise recording using the extended 1 Hz array might also increase the low energy content of the dispersion plot.

### Cross-hole tomography

The cross-hole tomography data were acquired using three boreholes in an L-shaped configuration. The shear wave source was located in the corner of the L, while the receiver string was located either at 10 m at the end of the short leg of the L or at 25–26 m at the end of the long leg of the L. Thus, the shots were performed twice: once recorded by the receiver array in the borehole of the short leg and once recorded by the receiver array in the borehole of the long leg. This geometry was chosen to generate tomographic images at two different scales and in two directions. The borehole source is coupled to the borehole wall by a pneumatic clamping system (inflatable bladder). The orientation of the source is controlled from the surface by a torsional stiff hose. The seismic blow direction was aligned perpendicular to the receiver borehole in order to generate SH-waves. To obtain the opposite blow direction the source was rotated by 180°, thus generating S waves with opposite polarities. For each shot direction a separate seismic record was acquired. The source also generated P waves.

**Fig. 12:**
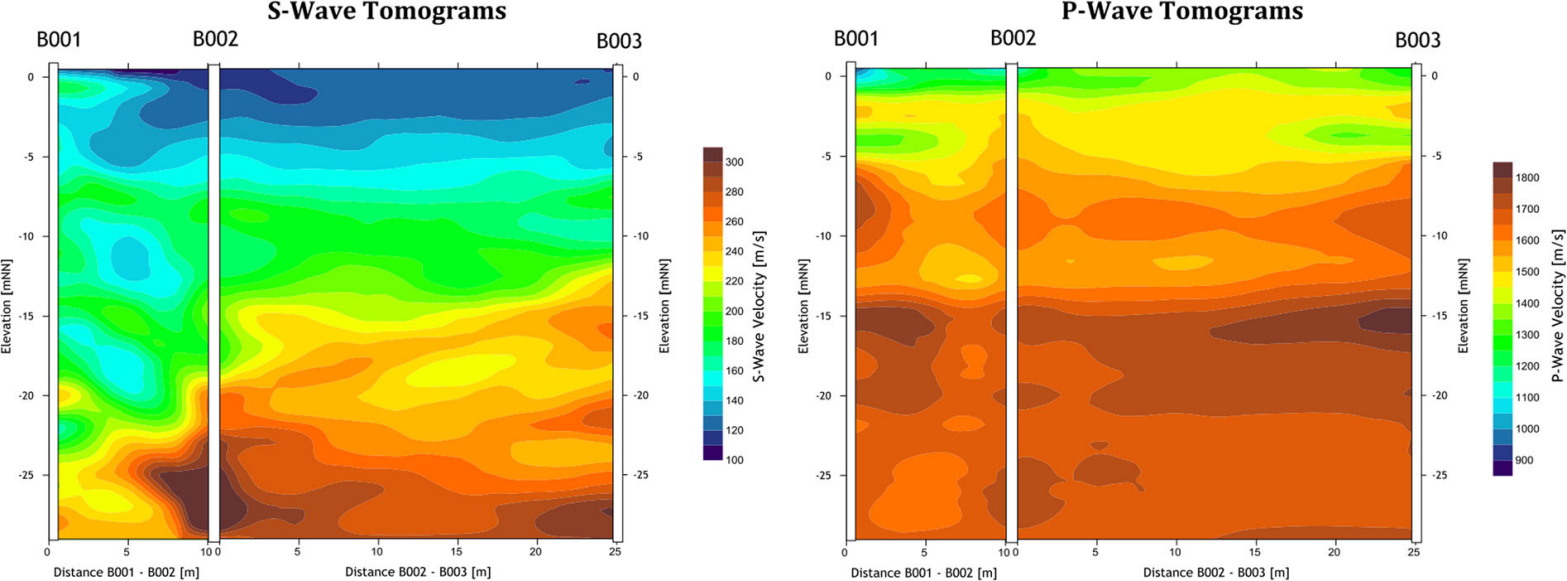
*V*_S_ and *V*_P_ images from cross-hole tomography for station BUHZ

The cross-hole seismic data processing included several steps. First, the travel times were determined manually by picking the arrivals of both the P and S waves. Next, the subsurface was numerically divided into cells. Vertical and horizontal cell sizes of approximately 1 m were selected. Seismic waves are assumed to propagate along curved ray paths. The simultaneous iterative reconstructive technique (SIRT; Gilbert, 1972) algorithm was used for travel time inversion. This algorithm is iterative and minimises the residual of the observed and calculated seismic travel times by a correction of the seismic slowness, i.e. the reciprocal of the seismic velocity in each cell. The tomograms for each borehole set were processed separately. An example of a *V*_S_ and a *V*_P_ tomogram is shown in Fig. 12. The heterogeneity of the sediment at short distance scales is apparent. The transition from relatively low (~ 180 m/s) to relatively higher *V*_S_ (250–300 m/s) at the Pleistocene surface occurs at ~ 14 and ~ 18 m depth in the two SCPTs at the location. The transition is present in the tomograms at a depth varying between 12 and 25 m, showing that the stratigraphy is variable even over distances as short as a couple of metres. Comparing the *V*_P_ and *V*_S_ tomograms shows that the *V*_S_/*V*_P_ or Poisson ratio is also highly variable over short distances and varies with depth.

### Seismic interferometry at vertical seismic arrays

In addition to the determination of detailed *V*_S_ profiles at the surface recording stations, interval *V*_S_ have been derived at ~ 70 near-surface vertical seismic arrays that cover the Groningen region (Fig. 1). The seismic arrays consist of geophones at 200, 150, 100 and 50 m depth and an accelerometer at the surface. Local events are recorded over this near-surface borehole network. Shear-wave velocities at depth levels where seismicity occurs are an order of 10 times larger than near the surface. Consequently, shear waves bend towards vertical propagation in the near surface and are largely recorded on the horizontal components. This illumination is suitable to estimate seismic interval velocities between the receiver levels in the boreholes. Two methods were considered to determine the interval velocities. The first method uses the time differences of single phases after correction for the angle of incidence. These single phases exhibit large uncertainties, both on the timing and on the angle of incidence. The second method, which is applied here, is seismic interferometry on a catalogue of local events with sufficient signal-to-noise ratio (Hofman et al., 2017). Horizontal-component seismograms are rotated towards the transverse component and cross-correlated over different depth levels of single boreholes. By cross-correlating entire waveforms and stacking in cross-correlations of many different events, a precise estimate is obtained of the local seismic response: the waveforms are obtained as if there were a seismic source at one of the receiver levels and all the other receivers measured its response. The obtained response is dominated by a direct up-going and down-going wave. From these waves, the timing is picked and converted to interval velocities along the boreholes.

## Integration of various methods

**Fig. 13:**
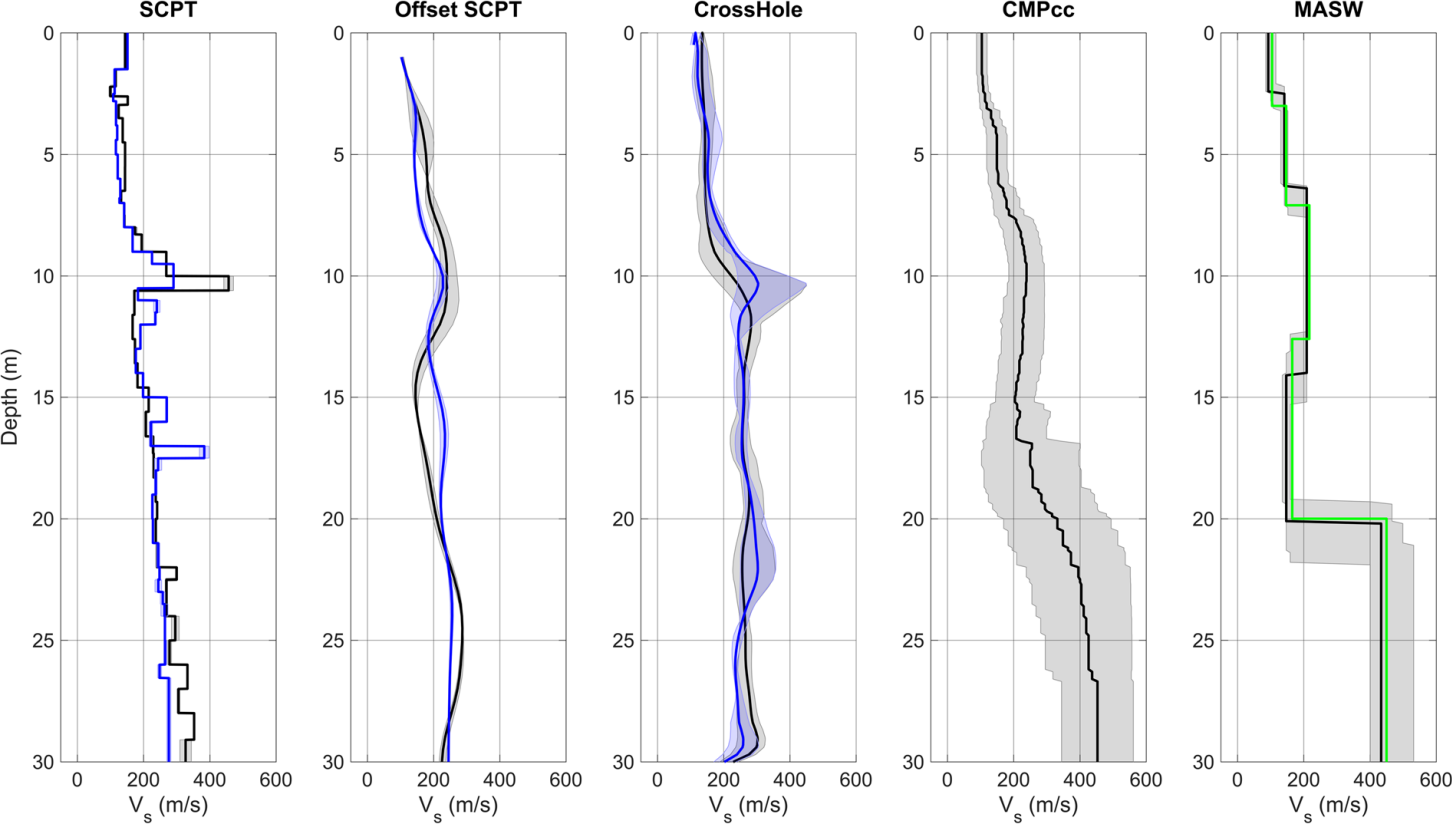
*V*_S_ profiles for station BWSE from different measurement techniques, including uncertainty bands. From left to right: SCPT03 (grey) and SCPT04 (blue) with band indicating variation between left and right blow; average profile for offset SCPTs between 1 and 5 m offset with shaded band indicating the minimum and the maximum *V*_S_; cross-hole average profiles for long leg (blue) and short leg (grey) with shaded band indicating the minimum and the maximum *V*_S_; CMPcc based on MASW array, shaded band indicates standard deviation of the 72 best models; MASW with manual fit in green and automatic inversion using the genetic algorithm in black with grey shaded band indicating the minimum and the maximum *V*_S_.

The various methods are based on different properties and behaviour of seismic waves and sample different kinds of soil volumes. All the results for one location are gathered and compared in Fig. 13. The sample volume of the methods and thus level of detail decreases from left to right. The SCPTs result in very detailed *V*_S_ profiles. However, they represent local *V*_S_ variations. The two SCPTs with 80 m offset near station BWSE are similar, but individual layers occur at slightly different depths and with varying *V*_S_ values. The *V*_S_ is low (~ 130–140 m/s) between the surface and ~ 8–9 m depth, next there is a faster layer of ~ 2 m (225–460 m/s) below which there is an increase in *V*_S_ from ~ 170 m/s to 350 m/s apart from a local high *V*_S_ layer at 18 m depth in SCPT04.

**Fig. 14:**
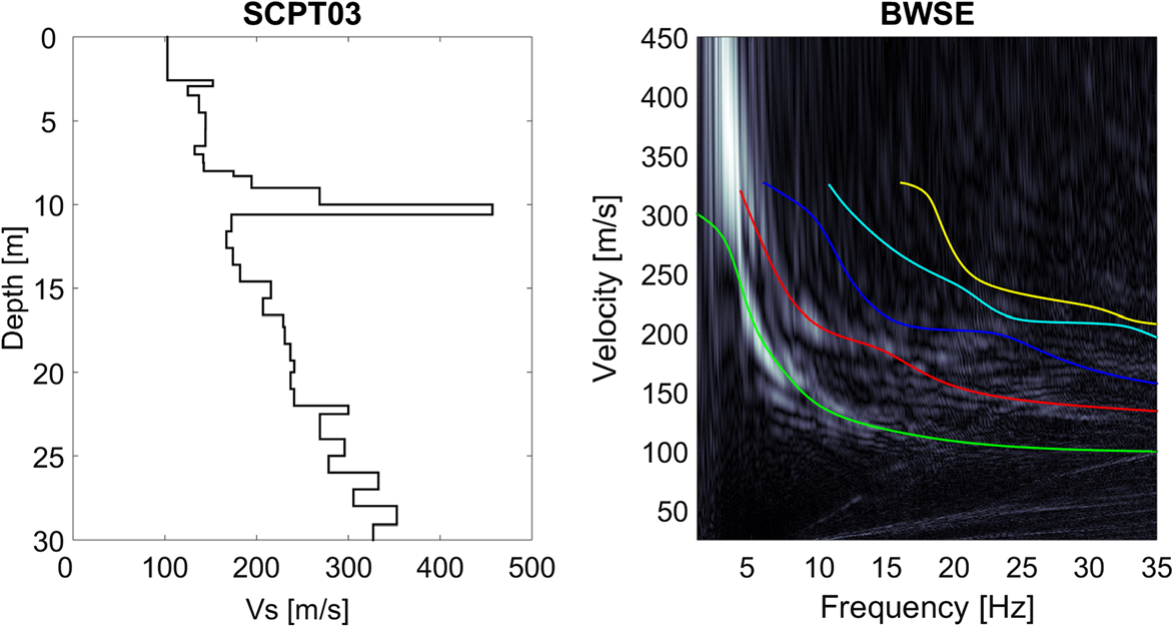
Example of SCPT profile on MASW for station BWSE. Left: SCPT03 *V*_S_ profile with the top 3 layers replaced by the minimum value. Right: theoretical dispersion curves of the *V*_S_ profile for fundamental (green) and higher modes (red, blue, cyan and yellow) plotted on top of the MASW dispersion plot (grey scale) for station BWSE

The original tomographic images of OSCPT and cross-hole *V*_S_ show the local variations in *V*_S_. In order to compare them to the other 1D profiles, they have been simplified by averaging *V*_S_ over depth slices. The 2D details, which are advantages of these methods, are lost in this way. The 1D OSCPT and 1D cross-hole profiles generally follow the SCPT profiles, but with less detail. The CMPcc and the MASW profile show a shallow *V*_S_ layer of ~ 90 m/s of 2 m at the top, followed by a 4 m thick layer of ~ 150 m/s, a 9 m thick layer of ~ 210 m/s and a 4 m thick layer of ~ 160 m/s. The very low *V*_S_ top layer in the MASW *V*_S_ profile is missed in the SCPT. Shear wave velocity results from the top 2–3 m in the SCPT cannot always be reliably determined due to the noisy record and the short distance of the waves travelled. This results in overlapping P and S waves in the record and the difficulty in reliably picking the S arrival. The gradual increase in *V*_S_ below ~ 11 m that is apparent in the SCPT was not resolved in the MASW *V*_*S*_ profile. The most representative *V*_S_ profile is a combination of the SCPT, cross-hole and the MASW *V*_S_ profiles with some weighing. For the present study, we adopted a pragmatic choice: the SCPT was combined with the top layer of MASW only if the SCPT results were unreliable for the top few metres. When two SCPTs were available, the station representative SCPT was chosen based on the distance between the SCPT and the station, the geology at the site and quality of the SCPT. To check the validity of the choice of SCPT, the theoretical dispersion curves of the SCPT were plotted on top of the MASW dispersion plot (Fig. 14). Because of the aforementioned possible unreliability of the top metres of the *V*_S_ from SCPT, the minimum *V*_S_ value of the top three layers was assumed for these layers. Generally, the fit is very good.

**Fig. 15:**
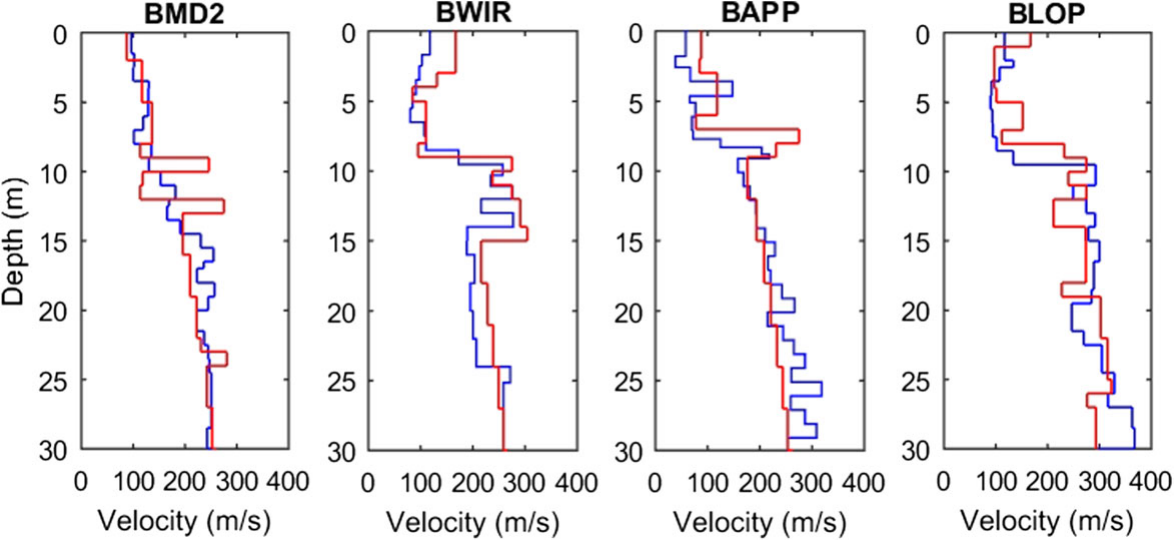
*V*_S_ profiles at selected B stations. Measured *V*_S_ in blue and modelled mean *V*_S_ (Kruiver et al., 2017a) in red

The 18 stations sample a variety of geological settings, but most stations are situated in areas with a Holocene cover on top of Pleistocene sediments. The average *V*_S_ is linked to the age of the deposits (Holocene or Pleistocene) and the lithology (peat, clay, sand). The transition between Holocene deposits (relatively low *V*_S_) and Pleistocene deposits (relatively high *V*_S_) is often easily recognised in the profiles by a jump from low *V*_S_ in the shallow layers to higher *V*_S_ values in the deeper layers (Fig. 15). This occurs at 11 m depth for station BMD2, at 9 m for BWIR, at 8 m at BAPP and at 9.5 m for BLOP. Individual peat layers can be recognised from their low *V*_S_, e.g. between 4.5 and 7 m at station BAPP. Between stations, the variation in *V*_S_ of the stratigraphic and lithological units is consistent. The general ranges of *V*_S_ for the Groningen deposits are < 100 m/s for Holocene peat, 100–200 m/s for Holocene clay and Pleistocene peat, 200–250 m/s for Pleistocene clay and fine sand and > 250 m/s for Pleistocene medium and coarse sand.

## Discussion

### Performance of techniques and sampling scales

The various techniques to determine *V*_S_ in the field generally perform well in the Groningen setting. The depth of penetration of SCPT is in most cases possible to the target depth of 30 m. In some cases, this maximum was not achieved because of a combination of high friction due to stiff clay, high tip resistance or high friction in the Pleistocene sands. The MASW suffered from limited depth of penetration in some cases as well. The most apparent case is station BAPP where the top 7 to 8 m consists of very low *V*_S_ (< 100 m/s) material. In this location, the dispersion could be determined down to approximately 1 Hz, but the modelling shows that it only contains information from the top 15 m.

The SCPT and cross-hole techniques are based on picking shear-wave arrivals. The source-receiver distance needs to be sufficiently large to be able to reliably distinguish between the arrivals of different waves. For the cross-hole setup, the distance between source and receiver was always sufficiently large for reliable first arrival picking. For the SCPT, on the other hand, the distance between the source and receiver varies with depth, because the source stays at the surface while the receiver penetrates the soil. As a consequence, the quality of the data from the SCPT varies with depth: the top part might be unreliable when wave forms overlap. The unreliable top part (2–3 m) of the SCPT *V*_S_ profile was replaced by the more reliable MASW results for three of the 18 stations.

We demonstrated that the resolution and scale at which the different methods obtain the *V*_S_ profile is quite different. The surface-wave method ‘averages’ the *V*_S_ information over the whole array length of approximately 200 m and therefore is only sensing *V*_S_ structures if they have a significant contrast, thickness and lateral extent. On the other hand, the SCPT method samples the velocity on a scale of a few metres at most. The OSCPT and cross-hole methods sample the data at intermediate scale. It is clear from the OSCPT, cross-hole and CMPcc results and the locations with two SCPT profiles that changes in velocities can be significant over small distances. Therefore, it is important to consider the relation between the scale of the *V*_S_ profile from a certain method and scale at which earthquake amplification occurs. The primary goal of the *V*_S_ measurements was to calibrate the transfer functions. A pragmatic choice was made to use the SCPT close to the accelerometer station (with MASW for the uppermost few metres when needed) for calculation, because it is both most detailed and contains extra information on lithology and shallow stratigraphy.

### Comparison to other types of *V*_S_ estimates and derived analyses

Additional to providing *V*_S_ profiles for the ground motion model, the SCPT *V*_S_ values were used to derive a *V*_S_ model for Groningen. For this, SCPT *V*_S_ from this fieldwork and from archive data were classified in terms of stratigraphy and lithology to derive *V*_S_ distributions (Kruiver et al. 2017a). These *V*_S_ distributions were used to model *V*_S_ in the entire Groningen field in the top 50 m using the detailed 3D voxel model of geology GeoTOP of TNO Geological Survey of the Netherlands (Stafleu et al., 2011; Maljers et al., 2015; Stafleu and Dubelaar, 2016). The modelled *V*_S_ profiles at the stations are shown in Fig. 15 for comparison with the in situ measured *V*_S_. There is generally good agreement between the measured *V*_S_ at the site and the modelled values, especially considering the regional character of the GeoTOP model.

**Fig. 16:**
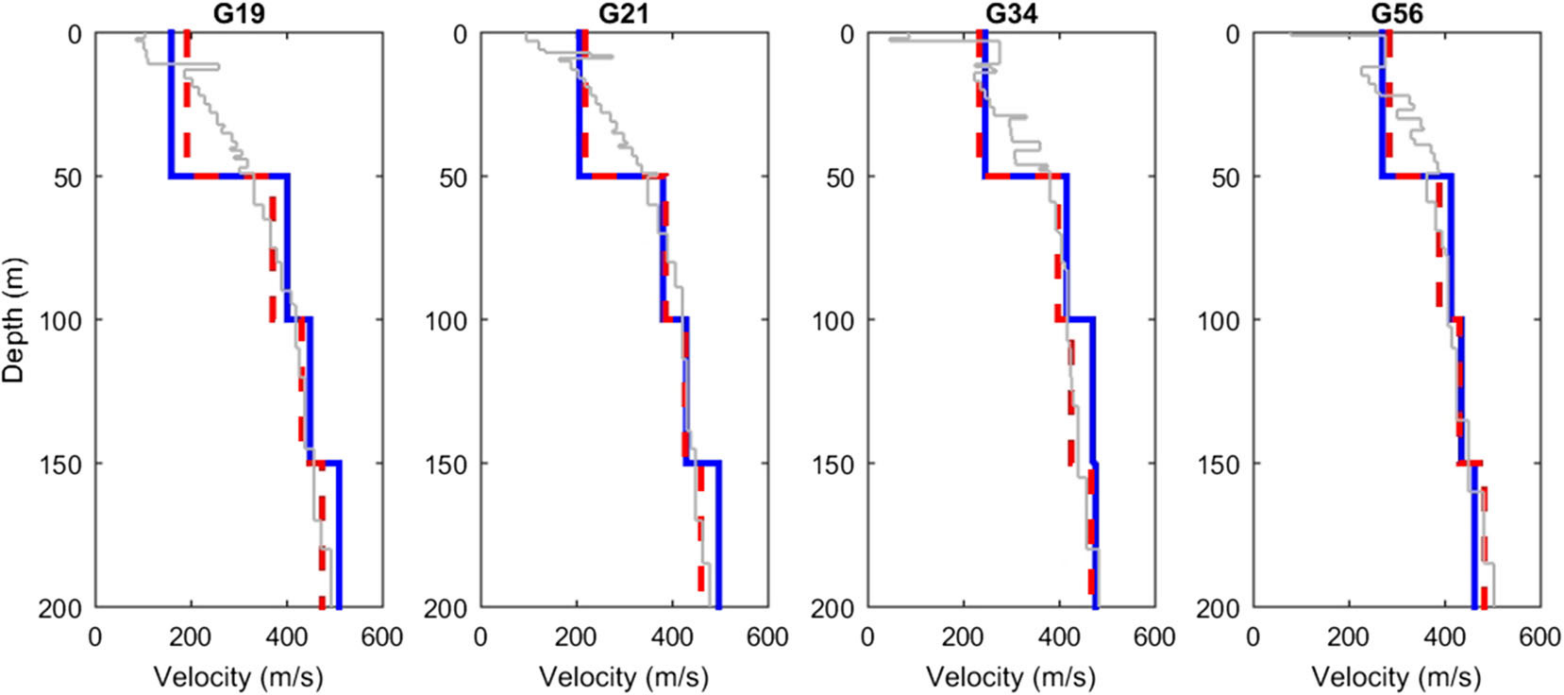
*V*_S_ profiles at selected G stations. Interval *V*_S_ from seismic interferometry in blue, modelled mean *V*_S_ (Kruiver et al., 2017a) in grey and harmonic means of modelled *V*_S_ in dashed red

Theoretical 1D SH-wave site transfer functions (TFs) were calculated for each of the measured *V*_S_ profiles at the B stations. These transfer functions were used to deconvolve Fourier amplitude spectra (FAS) of surface recordings to a reference horizon, at some 800 m depth, for the purpose of determining seismological parameters for modelling earthquake ground motions (Bommer et al., 2017). The simulated ground motions were then used in a non-linear soil response analysis using a field-wide velocity model (Kruiver et al., 2017a) to determine zone-specific spectral acceleration amplification functions (AFs) for use in hazard and risk analyses (Rodriguez-Marek et al., 2017). As such, it is important to verify both the consistency of the *V*_S_ models and the accuracy and applicability of the site TFs. At the G-stations from the KNMI monitoring network, no in situ measured *V*_S_ data are available. The *V*_S_ profiles inferred from seismic interferometry at selected G-stations and the modelled *V*_S_ profiles are compared in Fig. 16. The modelled *V*_S_ data have been converted to interval velocities corresponding to the geophone intervals by determination of the harmonic means. These compare very well to the interval velocities estimated from seismic interferometry. The main uncertainty in the velocities estimated with seismic interferometry is related to interference between the direct waves and the reflected phases and noise. In Hofman et al. (2017), confidence ranges are added to all estimated profiles. For *V*_S_, the confidence regions are generally very small and only exceed 20 m/s for a limited number of very noisy stations. Differences in the estimated and modelled *V*_S_ in the top 50 m can be attributed to local variations in geology at the station sites relative to the GeoTOP model.

Independent calculation of site transfer functions is typically performed using site-to-reference spectral ratios, or in the absence of a reference site (as in Groningen), by calculating empirical transfer functions from spectral modelling (Edwards et al., 2013). This approach has been successfully used in guiding the development of *V*_S_ models in Alpine and urban regions of Switzerland (Michel et al., 2014, 2016). The principal of empirical transfer functions is to isolate site effects. The approach of Edwards et al. (2013) uses a simple seismological point-source model (Brune, 1970; Anderson and Hough, 1984) to account for source and path effects in recordings of small earthquakes. Consistent site effects are then extracted from the intra-event FAS residuals over numerous events. By averaging over numerous events, distances and azimuths and extracting only the intra-event residuals, the non-uniqueness of spectral analysis approaches is largely removed (Goertz-Allmann and Edwards, 2013; Michel et al., 2014, 2016). The result is that site TFs are—over a broad frequency band—independent from the inverted source and path effects. Furthermore, in this analysis, we take advantage of the availability of the measured *V*_S_ as a priori information for the inversions and reduce possible trade-offs further. The 1D SH TF with vertical incidence from measured *V*_S_ profile is used as a starting model, with the inversion completely free to modify the TFs.

**Fig. 17:**
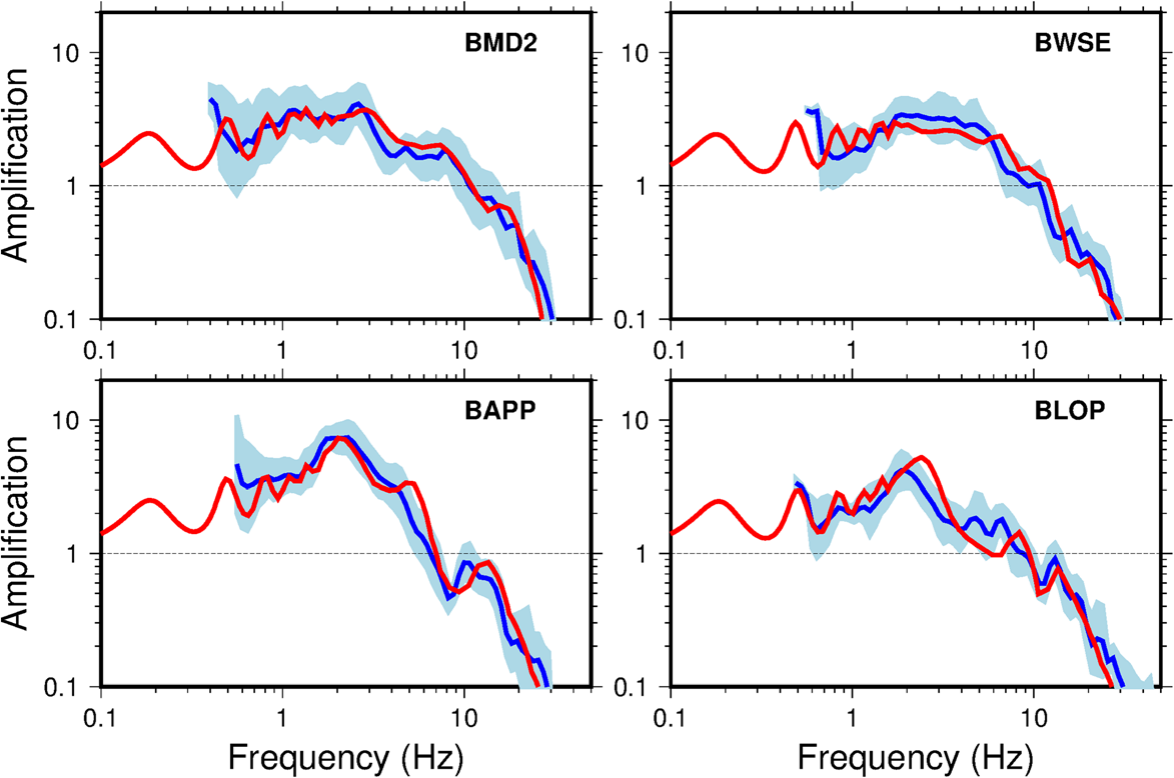
Comparison for selected B stations of empirical amplification (reservoir to surface amplification) from the Groningen earthquake recordings database (blue) and standard deviation (pale blue) along with the theoretical vertical 1D SH amplification between the reference and surface (red) based on numerical linear site response analyses

**Fig. 18:**
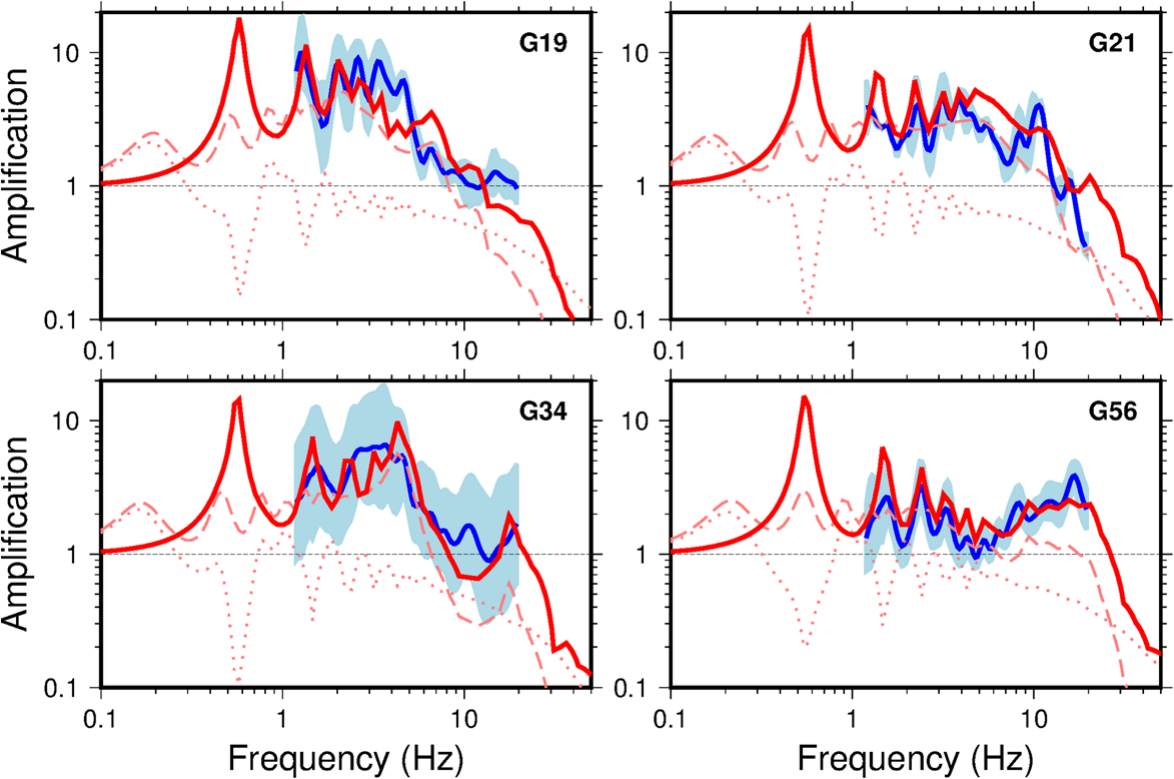
Comparison for selected G stations of FAS spectral ratio of surface to 200 m depth from the Groningen earthquake recordings database (blue) and standard deviation (pale blue) along with the spectral ratio of surface to 200 m depth from theoretical 1D SH linear site response analyses (solid red). The dashed red line indicates the 1D SH TF at the surface and dotted red line at 200 m depth

Figure 17 shows a comparison of the TFs for four sites with measured *V*_S_ profiles. The overall shape of the empirical TFs (determined from earthquake recordings following Edwards et al., 2013) and theoretical 1D SH TFs (determined from numerical linear site response analysis) is very similar, with only small differences in minor peaks and troughs. For the borehole (G) stations there are no measured *V*_S_ profiles. However, in this case, we can take advantage of the surface-to-borehole-at-200 m-depth (S/B) spectral ratio to calculate the effect of the soil column on the wavefield. Figure 18 shows S/B spectral ratios calculated using small earthquakes (1.5 > M_L_ > 3.1). In order to compare S/B spectral ratios with theoretical transfer functions, the TF between the borehole at 200 m depth (within-rock: i.e. accounting for both up- and down-going waves) and the outcropping surface must be calculated. This is equivalent to taking the ratio of the TFs between the reference horizon and both the outcropping surface and the 200 m depth ‘within-rock’ borehole levels. Generally, the amplification from the recordings and theoretical transfer functions agree well.

The good agreement between measured/inferred *V*_S_ and modelled *V*_S_ from Fig. 17 and Fig. 18 indicates that the use of vertical 1D SH TFs is appropriate over the 800 m from reference rock horizon to surface. The velocity models determined for the reference to surface produce remarkably similar TFs to those empirically observed in earthquake signals.

## Conclusions

A fieldwork campaign was conducted in the Groningen gas field to determine in situ *V*_S_ to approximately 30 m depth for the purpose of reducing uncertainty in the ground motion model for induced earthquakes. A suite of field techniques was used and existing techniques were extended. For example, the SCPT procedure was adjusted to take measurements at stratigraphy boundaries in order to sample each layer (especially peat) in sufficient detail and to avoid contamination of the measured *V*_S_ by the under- or overlying soil layer. We added source offsets to the SCPT (OSCPT) at 1 to 20 m from the cone location to be able to perform tomography of *V*_S_. This provided insights regarding spatial variations of *V*_S_ and representativeness of any individual SCPT.

The cross-hole tomography also showed that there is heterogeneity on spatial scales of one to several metres. For example, the jump in *V*_S_ associated with the transition between Holocene and Pleistocene deposits varies in depth by several metres over a horizontal distance of 25 m. The cross-hole tomography is very suitable to investigate spatial variation of *V*_P_ and *V*_S_ properties.

The MASW analysis consisted of both active and ambient noise data acquisition and various methods of processing. The classic MASW processing of the offset gather of the array resulted in the large scale *V*_S_ structures at the station sites. The passive and the MASW dispersion characteristics matched well. However, the microtremor array data did not significantly extend the bandwidth of the dispersion data relative to the MASW at our sites. This could be improved by including lower frequency geophones and ambient noise data collection for a longer period of time. The CMPcc approach on the MASW data indicated the lateral variability of *V*_S_ along the full array.

We made a pragmatic choice to achieve the final *V*_S_ profile at each station, using the SCPT, only substituting by the MASW value when the SCPT was unreliable. The comparison between the measured *V*_S_ and the modelled *V*_S_ that was used for site response analysis shows an excellent match. The correspondence between the *V*_S_ from seismic interferometry and the modelled *V*_S_ agrees very well. The measured *V*_S_ profiles significantly enhanced the ground motion model derivation by using them to deconvolve the recorded motions from the Groningen earthquake database to the reference baserock horizon.
